# Model interpretability on private-safe oriented student dropout prediction

**DOI:** 10.1371/journal.pone.0317726

**Published:** 2025-03-31

**Authors:** Helai Liu, Mao Mao, Xia Li, Jia Gao

**Affiliations:** 1 China Conservatory of Music, Beijing, People’s Republic of China; 2 University of Cambridge, Cambridge, United Kingdom; 3 China Development Bank, Zhengzhou, Henan, People’s Republic of China; Khalifa University, UNITED ARAB EMIRATES

## Abstract

Student dropout is a significant social issue with extensive implications for individuals and society, including reduced employability and economic downturns, which, in turn, drastically influence social sustainable development. Identifying students at high risk of dropping out is a major challenge for sustainable education. While existing machine learning and deep learning models can effectively predict dropout risks, they often rely on real student data, raising ethical concerns and the risk of information leakage. Additionally, the poor interpretability of these models complicates their use in educational management, as it is difficult to justify identifying a student as high-risk based on an opaque model. To address these two issues, we introduced for the first time a modified Preprocessed Kernel Inducing Points data distillation technique (PP-KIPDD), specializing in distilling tabular structured dataset, and innovatively employed the PP-KIPDD to reconstruct new samples that serve as qualified training sets simulating student information distributions, thereby preventing student privacy information leakage, which showed better performance and efficiency compared to traditional data synthesis techniques such as the Conditional Generative Adversarial Networks. Furthermore, we empower the classifiers credibility by enhancing model interpretability utilized SHAP (SHapley Additive exPlanations) values and elucidated the significance of selected features from an educational management perspective. With well-explained features from both quantitative and qualitative aspects, our approach enhances the feasibility and reasonableness of dropout predictions using machine learning techniques. We believe our approach represents a novel end-to-end framework of artificial intelligence application in the field of sustainable education management from the view of decision-makers, as it addresses privacy leakage protection and enhances model credibility for practical management implementations.

## 1. Introduction

Education is pivotal to the sustainable development of society [1–4], a fact underscored by the UNCED’s persistent advocacy for its significance. The UNCED posits that sustainable education exerts profound and enduring impacts across various domains, including the economy, ecology, and equity of all communities [1]. Consequently, enhancing students’ educational experiences and increasing the completion rates of higher education emerge as critical factors in fostering social sustainable development [3]. According to the Organisation for Economic Co-operation and Development (OECD), the dropout rate for higher education students was approximately 20% in 2019 [5]. In contrast, the United States had a much higher undergraduate dropout rate of 40% in 2020 [6]. The phenomenon of student dropout is multifaceted and have significant consequences on the individuals, university, and societal levels [7,8]. For instance, students who dropout from a higher education institution are more likely to remain unemployed for a longer period than those who complete their degree on time, leading to a waste of national investment in education [5,9]. Timely detection of students who are likely to drop out, and human teaching intervention to reduce the dropout risk of target students, have significant and positive social and economic implications. It has been reported that a significant improvement in micro-macro-economic returns can be achieved if the dropout rate in higher education decreases [9]. Consequently, scholars highly value this issue, and interest in student dropout prediction has been steadily increasing [10].

With the development of artificial intelligence, the use of various algorithms and models has become more widespread, and they have strong predictive capabilities. More and more scholars are using machine learning tools in the education fields [10,11]. As a result, datasets related to education, especially those related to students, have become a popular topic for data mining [12]. Moreover, the emergence of online class—the e-learning has opened up a stable channel for obtaining student data and student behavior data [13], Such as the Harvard, MIT’s online classroom EDX [14], and the Chinese version of MOOC, XuetangX [15]. An artificial intelligence competition also launched at the using the educational dataset built by Knowledge Discovery in Databases(KDD) [16], to predict whether students can complete their selected courses by analyzing online behavior. For university education management, obtaining online data from students can be challenging, and online activities are not the primary behavioral component of on-campus based students. However, research on data samples composed of student on-campus behavior and characteristics is still relatively in small size [17] and the cost for obtaining such data is usually high [16].

On-site data, which reflects students’ realistic features and characteristics, is highly sensitive and private. The intentional, unintentional, or inappropriate disclosure of this private information can have adverse societal impacts [18,19]. Generative Adversarial Networks (GANs) [20] and its derivatives, which can generate synthetic data, offer a promising solution to this issue [21,22]. Additionally, the emerging technique of dataset distillation [23], which involves creating synthetic datasets with much smaller size compared to the original data, while retaining the essential characteristics of the original data, is also a viable option for protecting student privacy.

From the perspective of educational management, model interpretability is crucial for decision-making. Understanding the specific reasons behind predictions allows educators to provide personalized help to at-risk students [24], such as remedial courses, and tutoring sessions [25]. This enhances the trust and applicability of the models in educational settings [25]. Given the complexity of machine learning models, it is essential to explain the specific parameters that affect student dropout. Students are more likely to accept well-explained indicators that reflect their risk of dropping out, as only well-understood variables can be used for effective tutoring guidance. Aligning the interpretability of features with the logic of educational management is of essential benefits [17]. This article, from the perspective of a higher education decision makers, focuses on the privacy protection and interpretability of machine learning models and assigns educational significance to each parameter. We conducted in-depth research and analysis on tens of indicators affecting student dropout and provide a potential protocol for higher education administration. The main contributions of this study are listed as follows:

- Firstly, we focused on on-campus educational management purposes and build up a novel end-to-end student dropout prediction framework, to utilize data reconstruction techniques, such as the conditional generative adversarial networks and the Kernel Inducing Points (KIP) data distillation, to generated new samples that simulating real students information, preventing private information leakage, and followed by model explanation from both quantitative and qualitative view, to enhance the model credibility in the eye of education decision makers and students.
- Secondly, we introduced a novel data preprocessor to the KIP dataset distillation technique, and formed the PP-KIPDD algorithm for tabular dataset distillation purposes and reconstructed the student simulated datasets. Our approach demonstrated a better simulation and performance compared to the traditional data synthesis technique--the Conditional Generative Adversarial Networks. This method aims to prevent student privacy leakage during most of the machine learning process. To the best of our knowledge, this is the first time to use dataset distillation protocol in the field of student dropout prediction application and thus prevent student information leakage.
- Thirdly, we enhanced the model credibility by combining the quantitative explanation of machine learning with a straightforward explanation from education management aspect. In detail, we applied SHAP (SHapley Additive exPlanations) analysis to express model interpretability. We then conducted an in-depth study of the selected variables to determine if the features were well-explained from educational perspectives. This analysis demonstrated that these variables offer practical insights for on-campus educational management and have the potential to prevent student dropouts in advance, thereby assisting the educational management decision-making process.

### 2. Literature review

Numerous studies have been conducted, utilizing either qualitative analyses [26–29], empirical analyses [30–34], or machine learning techniques [11,35–38], to identify factors that might be influential for student dropout or to develop protocols to predict student dropout. These studies provide meaningful and useful information for the policy-making process. S. J. Greenland and his group [26] utilized questionnaires and interviews for qualitative analysis and identified five intervention themes and 19 sub-themes that reflect students’ opinions on what might have helped them complete their studies. They provided dropout intervention information to prevent students from dropping out of open online classes. Xavier *et al*. utilized content analysis of in-depth interviews with 16 undergraduate learners and focused on how time challenges impacted their decision to withdraw the classes [28]. Regarding statistical analysis, W. Li’s group conducted a deep analysis to identify factors that influence student dropout by utilizing empirical analysis. In addition to increasingly sophisticated methods for analyzing the correlations of various factors for student dropout, new dimensions of behavioral data have also been introduced for evaluating student dropout prediction. The analysis data on student dropout contains unprecedented richness. For example, the main features from research focusing on improving the dropout prediction of MOOC users are typically students’ clickstream data extracted from e-learning platforms, which is quite different from traditional samples derived from designed polls and questionnaires [39,40]. In line with the development of big data, the progress of machine learning has also brought new ideas to the field of educational technology. Compared with traditional methods, using machine learning models to conduct research in education can cover a wider range of factors and more samples. C. H. Cho compared several machine learning protocols and considered using re-sampling techniques to enhance model performance [41]. W. Xing and his group constructed a dropout prediction deep learning model based on temporal mechanism, which outperformed the baseline models’ performance [42]. It has also been reported that training auto-encoding adopted LSTM network will increase the prediction accuracy and reduce overfitting on the low-performing students group [43]. Zhao et al. used different models to train different features and fused the sub-output to improve the model performance [44]. An increasing number of researchers are introducing artificial intelligence techniques into the field of preventing student dropout. Although most of them use existing machine learning models to train the samples, this may be due to the fact that students’ dropout behaviors depend on teaching forms and cultures from different countries.

Despite the advancements in machine learning for student dropout prediction, privacy issues arise as machine learning typically uses real student information. The intentional or unintentional leakage of students’ private information can lead to various social problems, such as telecom fraud and unsolicited study abroad service recommendations. The issue of privacy has garnered increasing attention from scholars, with one popular approach being data synthesis based on original real samples. During model training, only synthetic data is provided, which helps protect individual student privacy. Among data synthesis methods, Generative Adversarial Networks (GANs) [20] is known for its superior capacity for mimic the real data distributions. And in terms of the tabular data structures of school students, Conditional GAN (CGAN) [45] is of great potential, which is specializing in tailoring with tabular datasets. Additionally, the recently developed dataset distillation is another effective choice. Dataset distillation is a process that generates synthetic data containing rich spatial patterns representing the original dataset’s information [23,46] which was known for its great potentials in privacy protection [22], was first introduced by Wang et al., [47] and has been showing receiving increasingly interests by researchers during recent a few years. Typically, the performance of models on a small distilled dataset can approach that on the original dataset. Among various number of distillation techniques, Reference [48] (the KIPDD method) is a kernel ridge regression based approach, which belongs to a performance matching branch of distillation technique. However, the KIP dataset distillation was developed using image samples (3-channels or 2-channels with grids), which is often different in nature from the tabular structured datasets (1-channel). Distillation techniques for tabular dataset is still not seen much in research [49], leaving this challenge to be solved for the purposes of privacy protection in student information.

In addition to privacy concerns, from an on-campus educational management perspective, schools also focus on model interpretability. The quality of interpretability directly affects the trust that educational decision-makers place in the model and can provide personalized help to students at risk of dropping out, thereby preventing them from leaving school. Consequently, research on model interpretability [50] has become a hot topic in recent years. SHAP (SHapley Additive exPlanations) [51] value analysis offers a robust solution for model interpretability. SHAP values focus on both global and local interpretability analysis. They not only explain the importance of each feature in the context of all features for the model’s prediction but also quantify the significance of local variables for individual sample predictions. Therefore, using SHAP values can provide reliable evidence for educational administrators’ decision-making.

Thus, in this article, we first solved the problem of distilling tabular dataset, by introducing a preprocessor to KIPDD. We considered the practical scenarios of on-campus educational teaching management and reconstructed the dataset using our proposed method, compared with CGANs. We then applied SHAP values [52] to select the most explainable features in the learning process. A combined reasoning deduction was followed up to show the features’ generality and rationality.

## 3. Materials and methods

**Fig 1:**
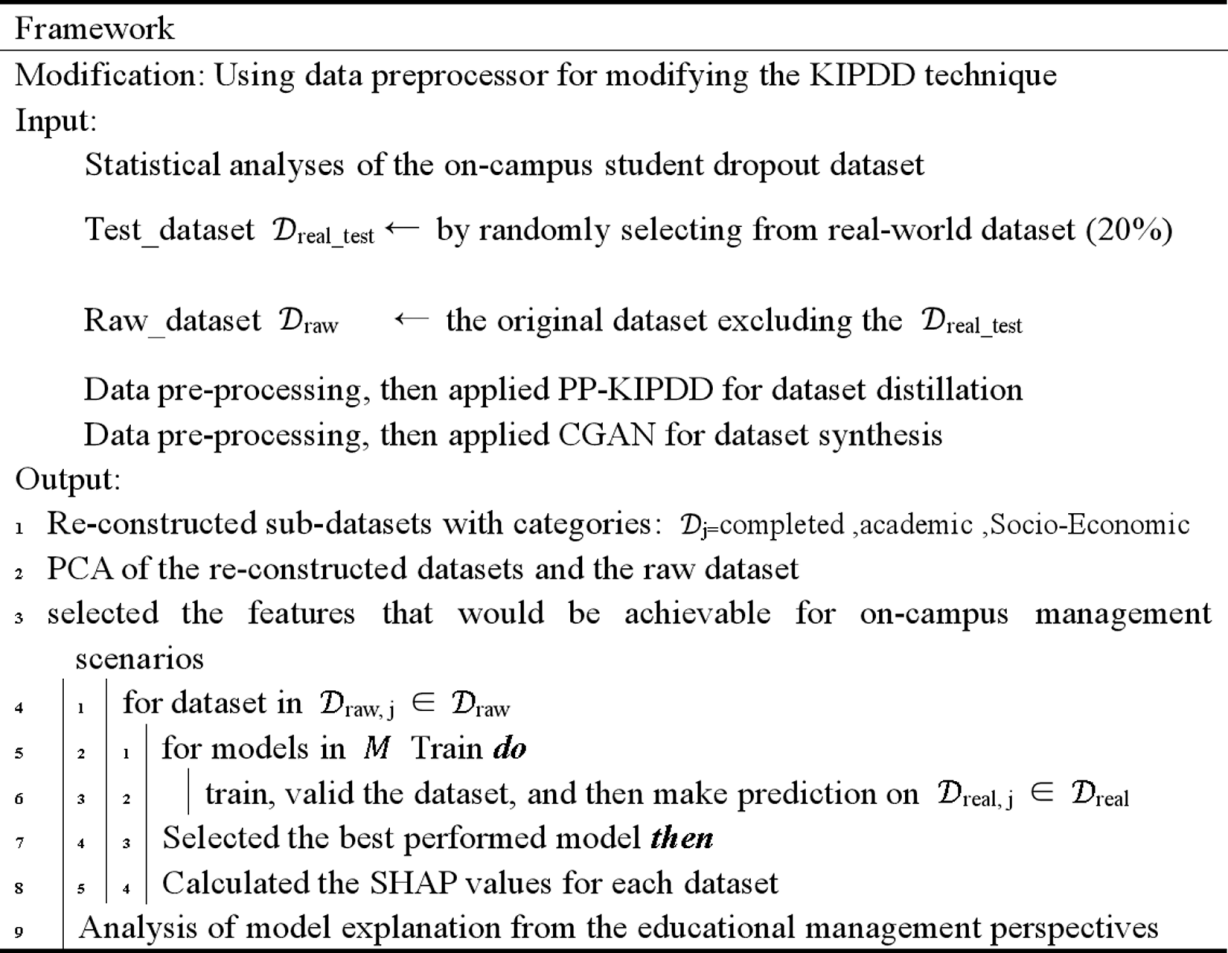
Framework of this article. Data redistribution was carried out after the statistical analyses. Various machine learning techniques including SMOTE-LightGBM were applied for student dropout predictions. Interpretability was evaluated using SHAP values and the connections between machine learning Interpretability and educational management practices were bridged.

In this article, a descriptive data analysis was conducted to determine the distribution of each feature, and the definitions of these features were also provided. It was recognized that the features could be categorized into various aspects such as macroeconomics and student academic performance, leading to the reconstruction of several sub-datasets from the raw dataset. We then employed Conditional Generative Adversarial Networks and Kernel Inducing Points dataset distillation techniques to create two sets of synthetic data representing the spatial information of the original data, thereby avoiding privacy leakage (the dataset created by CGAN is denoting to **Synthetic Dataset**, while the dataset created from distilled technique, is denoting to **Distilled Dataset**). Subsequently, we applied several principal machine learning protocols to train and predict on the various synthetic datasets and identified the best-performing models. The SHAP values of the best model were also calculated, visualized, and their educational significance was discussed in detail. The framework of this article can be summarized in Fig 1:

### 3.1. Dataset description

We utilized data that described student academic performance with several aspects, including macroeconomic, socioeconomic, and demographic and on-class academic characteristics. The dataset was collected from higher education institutions (acquired from several disjoint databases). On-Class academic performance includes features such as the number of curricular units approved/credited/enrolled by the student in the first or second semester. Macroeconomic information includes GDP, inflation rate, unemployment rate. Socioeconomic features describe students’ tuition fees up to date, debtor and scholarship holder status, and demographic characteristics measure students’ gender, age and others. Table 1 provides a brief description of data distribution and categories. As the dataset contains no null values, and all categorical data has been encoded to numerical data, we applied the dataset as it stands.

**Table 1:** Data descriptions of all features, the mean values, standard deviations, min and max values were demonstrated, and all features were categorized.

Variables	Mean	Std.	Min	Max	Category
Marital status	1.18	0.61	1.0	6.0	Demographic
Daytime/evening attendance	0.89	0.31	0.0	1.0	
Nationality	1.25	1.75	1.0	21.0	
Mother’s qualification	12.32	9.03	1.0	29.0	
Father’s qualification	16.46	11.04	1.0	34.0	
Mother’s occupation	7.32	4.00	1.0	32.0	
Father’s occupation	7.82	4.86	1.0	46.0	
Gender	0.35	0.48	0.0	1.0	
Age	23.27	7.59	17.0	70.0	
Displaced	0.55	0.50	0.0	1.0	
Unemployment rate	11.57	2.66	7.6	16.2	Macro-economic
Inflation rate	1.23	1.38	-0.8	3.7	
GDP	0.00	2.27	-4.1	3.5	
Curricular units 1st sem (credited)	0.71	2.36	0.0	20.0	Academic
Curricular units 1st sem (enrolled)	6.27	2.48	0.0	26.0	
Curricular units 1st sem (evaluations)	8.30	4.18	0.0	45.0	
Curricular units 1st sem (approved)	4.71	3.09	0.0	26.0	
Curricular units 1st sem (grade)	10.64	4.84	0.0	18.9	
Curricular units 1st sem (without evaluations)	0.14	0.69	0.0	12.0	
Curricular units 2nd sem (credited)	0.54	1.92	0.0	19.0	
Curricular units 2nd sem (enrolled)	6.23	2.20	0.0	23.0	
Curricular units 2nd sem (evaluations)	8.06	3.95	0.0	33.0	
Curricular units 2nd sem (approved)	4.44	3.01	0.0	20.0	
Curricular units 2nd sem (grade)	10.23	5.21	0.0	18.6	
Curricular units 2nd sem (without evaluations)	0.15	0.75	0.0	12.0	
Educational special needs	0.01	0.11	0.0	1.0	Socio-economic
Previous qualification	2.53	3.96	1.0	17.0	
Scholarship holder	0.25	0.43	0.0	1.0	
Debtor	0.11	0.32	0.0	1.0	
Tuition fees up to date	0.88	0.32	0.0	1.0	
International	0.02	0.16	0.0	1.0	
Application mode^*^	6.89	5.30	1.0	18.0	
Application order^*^	1.73	1.31	0.0	9.0	
Course^*^	9.90	4.33	1.0	17.0	

* As application mode, application order and course information were assigned to students before any academic performance occurred, this article categorized these three factors into Socio-economic aspect for convenience.

### 3.2. Feature correlation analysis

In this dataset, there are three statuses for a student attending the courses: dropout, enrolled, and graduated. We set the enrolled and graduated statuses as non-dropout of the course and correspondingly, we set the target who dropped out under this circumstance as 1. Thus, the scenario becomes a binary classification problem, where the target equals 0 means the student finished their class, while the target equals 1 means a dropout behavior.

**Fig 2:**
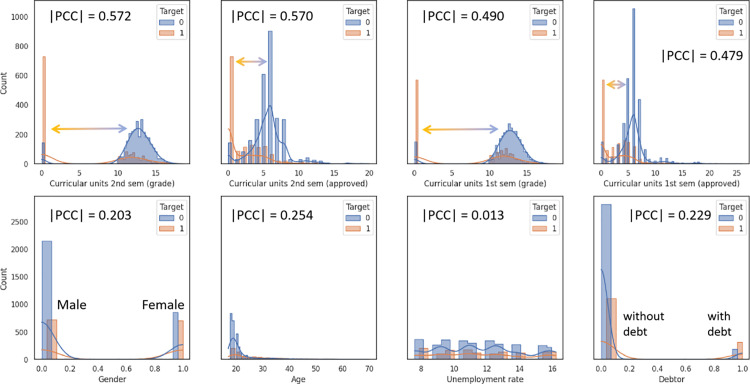
PCC values and the histogram of selected features towards the target. First row represents the features from the Academic Category. The second row demonstrates features from Demographic Category and Macro-economic Category.

We first overview the correlations between raw variables, where all variables’ correlations were measured by Pearson Correlation Coefficient (PCC). Regarding to the model prediction target (whether student dropout or complete their course), we visualized several selective variables towards the Target, as seen in Fig 2. The **| PCC |** values of students’ academic performance (Academic Category) usually share high **| PCC |** values and large standard deviations. Variables in the first row of Fig 2 represent the students’ academic performance information, the absolute values of PCC towards Target are obviously higher than the variables in the second row, which measure students’ demographic information or the macro-economic status. Curricular Units 2nd Sem (grade) and Curricular Units 2nd Sem (approved) reached 0.572 and 0.570, respectively, while the **| PCC |** value of demographic information shown in Fig 2 range from 0.013 to 0.229. Though highly correlated features provide useful information, it has been regarded a high **| PCC |** value might lead to model overfitting and instability problems, as the strong collinearity between the trainset and the target is supposed to leak the target information [53–56]. The histogram in Fig 2 also give clues of the information leaking: in the first row, when the value of Curricular Units 2nd Sem (grade) below 2, most students tend to drop their classes, while when the values is more than 10, most students are marked as completed their classes. The different distributed peaks between dropout students (target =  0) and non-dropout students (target = 1) indicated the possibly leakage of target information. Moreover, highly correlated features were also limited the trained models’ generalization [56].

**Fig 3:**
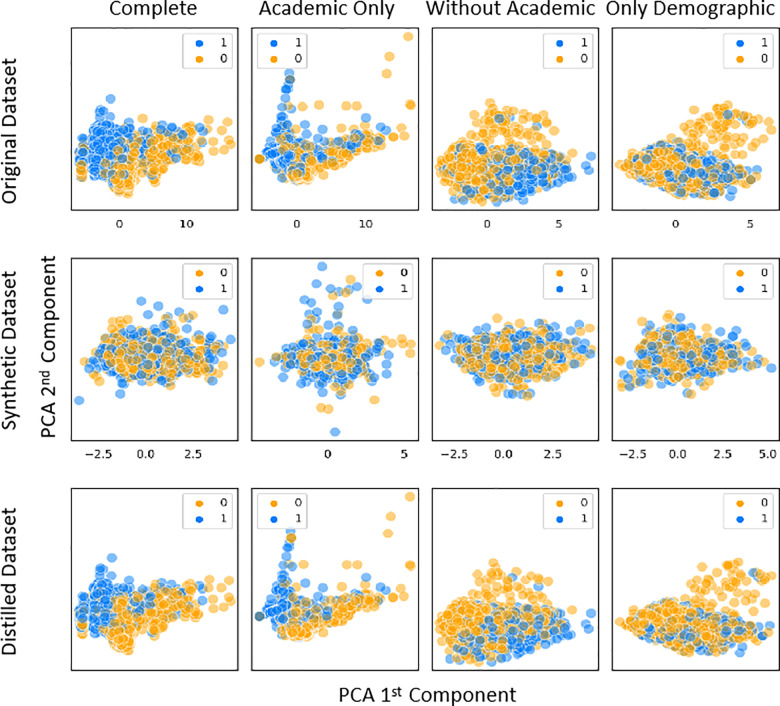
The PCA visualization of sample distribution of student with academic performance *vs* student without academic performance. The x-axis and y-axis represent the 1st and 2nd component in PCA.

Academic performance is intuitively correlated to students and to some degree determine whether a student will dropout or not. It is one of the most important information to evaluate students’ performance across the whole period during school. Moreover, the academic information (grades) share following advantages, 1) the rich of data source as each university can easily access their data, and 2) it helps tutors to know the students academic features thus design personalized enrollment guidance and orientations and such application has been successfully implemented by recent research [11] Fig 3. Academic Only distribution plot also indicates a more separable boundary can be seen compared to dataset without academic information. However, tutors cannot obtain this data at the beginning of a student’s academic journey, and thus this information cannot demonstrate the very first information to help tutors prevent students from dropping out of school. Thus we constructed several sub-datasets from the raw dataset: the complete dataset (raw dataset), dataset with academic information only (only academic dataset), and the dataset without students’ academic performance (selected dataset). We also utilized Principal Component Analysis (PCA) analysis, to project to features to lower dimension space, to have a glance at the distributions of these sub-datasets, which can be seen in Fig 3. (the Original Dataset, the first row).

The change in distribution is obvious with and without the highly correlated features (the academic performance of students). The upper left image represents the original data profile, where it is clear to classify the students who are going to dropout (the blue dots) and the students who are going to complete their degrees (the orange dots). When the academic features are excluded, the samples become harder to classify compared to the original one. Although the sample distribution on the right-hand side represents a more hard-to-classify scenario, it is more meaningful than the left one discussed above. Therefore, we are going to utilize the constructed sub-dataset intentionally excluding student academic performance data for discussion.

### 3.3. Dataset distillation

we first split the datasets to raw dataset, *D*_raw_, for model training, and real dataset, *D*_real_test_, for model test with a ratio of 80%: 20%. The *D*_real_test_ was used only for model prediction in order to prevent any information leakage during dataset synthesis process.

We employed two methods for data synthesis: Conditional Generative Adversarial [23] Networks for data synthesis and the modified Preprocessed Kernel Inducing Points data distillation (PP-KIPDD) technique for dataset distillation. The CGAN specializes in tailoring structured data, such as tabular datasets. It leverages the power of GANs to learn the underlying distribution of real tabular data and generate new, realistic samples. This is particularly useful in scenarios where data privacy is a concern or when the available dataset is limited. CGAN consists of two main components: the Generator (G) and the Discriminator (D). The Generator aims to produce synthetic data that is indistinguishable from real data, while the Discriminator attempts to differentiate between real and synthetic data.

Kernel Inducing Points [48] is a performance matching dataset distillation technique that leverages the principles of kernel ridge regression that targets on optimizing synthetic datasets, and model trained on those datasets will obtain minimized loss on the original dataset. The model performance on synthetic and on real datasets is thus matched [23]. As there has been no report about the use of KIP dataset distillation in tabular dataset, we first modified this technique, improving its capacity in tailoring with tabular dataset, by modify the input dimensions. Other hyperparameters were kept the same as the original method.

As KIPDD is developed using image dataset, which is different in nature with tabular dataset in this article. Our modification on KIPDD mainly focuses on re-structuring the datasets. In detail, the tabular data is 1-channel, which is different from that of image data (2-channel or 3-channel). The original KIPDD suits for processing 3-channels image data, and uses ReLU activation for performance matching. PP-KIPDD up-scaled the tabular dataset to a 3-channelled datasets, and filling up NAN with 0. As this is a performance matching approach, a Cross-Entropy Loss Function should be applied in order to fit the dropout prediction (a binary classification scenario). However, as binary classification usually represents less information than the mean-square-error, in this article, to better simulate student information distribution, we applied MSE as the distillation loss function, instead of using Cross-Entropy. The PP-KIPDD is then built up buy the embedding of the data-preprocessor. In the following of this article, the dataset distilled from PP-KIPDD is represented as “distilled data”, and the dataset synthesized through CGAN, is shown as “synthetic data”.

**Fig 4:**
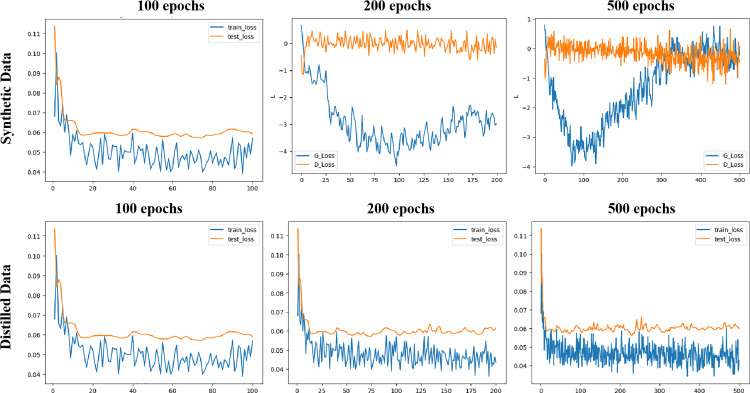
The learning curves varied depending on training epochs. The G-loss and D-loss converges after 500 epochs for the CGAN algorithm, while the training loss and test loss of distilled dataset converges after only 100 epochs.

Dataset reconstruction was carried out using a GPU-100 graphics card with a memory of 16 GB. First, we demonstrate the relationship between the learning curve and training epochs, as shown in Fig 4. For the CGAN method, the performance at 100 epochs was relatively average. However, at 200 epochs, there was a noticeable improvement in the learning curve, and by 500 epochs, the learning curve had reached convergence. In contrast, for the dataset distillation method, the learning curve remained stable at 100, 200, and 500 epochs. This indicates that dataset distillation achieves a stable and converged state as early as 100 epochs, demonstrating higher efficiency compared to the current CGAN method.

### 3.4. Model training

We applied traditional machine learning models for dropout prediction, including logistic regression, KNN, random forest, LightGBM on the raw dataset, and the selected dataset (without the academic information). It is to notice the ratio of dropout students (Minority) over non-dropout students (Majority) is 0.38, which is slightly imbalanced distributed. The imbalanced distributed datasets might lead to a bias towards the majority class during the training process [57]. To evaluate if such imbalanced distributed data will influence the model performance, Synthetic Minority Over-sampling Technique (Smote) [58] was also applied for predicting students dropout behaviors on those three sub-datasets.

### 3.5. Evaluation metric

The receiver operating characteristic (ROC) curve is a widely used tool in binary classification problems to evaluate the performance of a model or algorithm and especially for imbalanced distributed datasets. It is a plot of the true positive rate (TPR) against the false positive rate (FPR) for different threshold values. The area under the ROC curve (AUC) is a single number that summarizes the overall performance of the model performance. AUC ranges from 0 to 1, where 0.5 indicates a random guess and 1.0 indicates a perfect classifier. AUC is a popular metric because it is threshold-agnostic and provides a comprehensive summary of the model’s performance over all possible thresholds. Besides, F1-score and ACC are commonly used metrics to evaluate the performance of binary classification models, such as this scenario of student dropout prediction. Therefore, in this article, we are going to apply these values for model evaluation. In detail, the metrics above are based on different aspects of the confusion matrix, which summarizes the true positives (TP), false positives (FP), true negatives (TN), and false negatives (FN) of the model predictions.

Precision is the ratio of true positives to the total number of positive predictions. It measures how many of the predicted positive cases are actually positive. The formula for precision is:


$$\text{Precision}=\frac{\text{True Positives}}{\text{True Positives }+\text{False Positives}}$$

Recall (TPR) is the ratio of true positives to the total number of actual positive cases. It measures how many of the actual positive cases were correctly predicted. The formula for recall is:


$$\text{Recall }\left(\text{TPR}\right)\text{=}\frac{\text{True Positives}}{\text{True Positives }+\text{False Negatives}}$$

Accuracy measures the correctness over all samples and its formula as follows:


$$\text{ACC }=\frac{\text{True Positives }+\text{True Negatives}}{\text{True Positives }+\text{True Negatives}+\text{False Positives }+\text{False Negatives}}$$

F1-score is the harmonic mean of precision and recall. It is a measure of the balance between precision and recall. The formula for F1-score is:


$$\text{F1-score}=2*\frac{\text{Precision * Recall}}{\text{Precision }+\text{Recall}}$$

### 3.6. Model explanation

Student performance on courses is crucial [59]. Many studies have shown that students’ personal effort has a direct and significant impact on dropout rates [7,60]. They concluded that academic performance-related features (such as curricular units enrolled, credited, evaluations, approved, and grade, *et al.*) are the best explanation for the model and are the most important factors in terms of correlation and feature importance. This is consistent with the output results of our model. However, due to the strong randomness of academic performance (such as different students performing differently in different types of courses), we believe that these academic performance features will limit the model’s generalization ability. Furthermore, the academic performance can be regarded, in some extent, the reason of dropout behaviors thus there might be information leakage for model training. The third drawback of using academic performance as the training features is that in practical educational management, information prior to university entrance is an effective way to prevent students from dropping out. Therefore, we removed these academic performance-related indicators and conducted a new round of training and learning on the dataset using the machine learning models mentioned earlier. The model outputs showed that the AUC-ROC of all models decreased. Considering LightGBM has a general strong performance on tabular tasks, we started from LightGBM and conducted research on the interpretability of features that excluded student academic performance from an educational perspective. These features can be divided into several aspects, such as economic factors (GDP, inflation ratio, debt), personal factors (gender, age), and student social attributes. We will analyze them one by one in the following section.

## 4. Results

Without loss of generality, we classified student features according to ***Academic***, ***Demographic***, ***Socioeconomic***, and ***Macroeconomic*** factors and selected several relatively important indicators from each category for analysis. We mainly discuss the features that are significant in the datasets without academic information. This is because such features are more achievable and generalized in practical educational management.

### 4.1. Datasets synthetic properties and model performance

The results indicate that dataset distillation is more suitable for generating student data to be used as a training dataset without leaking personal information, from both construction efficiency and model performance of prediction power. Fig 3 demonstrated the data distribution profiles of the original dataset (first row, details can be seen in 3.2 Feature Correlation Analysis), the synthesized dataset (second row) and the distilled dataset (third row). Through it seemed the training process was converged (Fig 4) for CGAN synthesis, the distribution profiles showed an obvious distortion to that of the original raw dataset’s distribution profiles. In comparison, the profiles of the PP-KIPDD distilled datasets demonstrate a visually similarity to that of the original datasets, indicating a better mimic capacity. This conclusion is also supported by the model performances on those datasets, where in most cases, AUC scores achieved from PP-KIPDD distilled datasets are more close to the original dataset.

**Fig 5:**
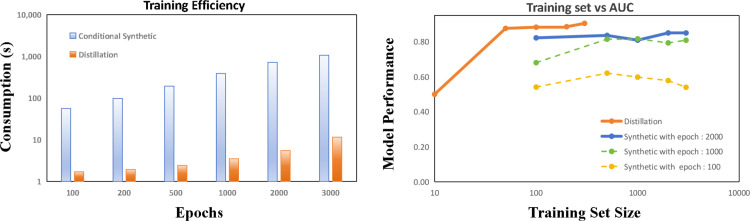
The training efficiency (left-side image) and the model performance (right-side image) of the dataset synthetic process. PP-KIPDD distillation only takes less than 10 seconds for 2000 epochs, compared to that of CGAN using about 1000s for 2000 epochs of training. The model performance on training sample sizes showed the model (LightGBM) performance on distilled dataset is better than that on CGAN generated datasets.

Firstly, to compare the effectiveness and efficiency of data synthesis, we studied the training epochs and computational costs. As shown in Fig 5, **the Dataset Distillation technique is faster and shows good training convergence.** We selected epoch values of 100, 200, 500, 1000, 2000, and 3000 to test the computational costs on GPUs for these two algorithms. As depicted in Fig 5, it is evident that the CGAN technique has lower efficiency, meaning higher running costs that increase exponentially. For instance, at 100 epochs, CGAN’s running time is over 90 seconds, whereas dataset distillation only requires a few seconds. With an increasing number of epochs, CGAN’s running time rises exponentially; at 3000 epochs, its running time exceeds 2000 seconds, while dataset distillation only requires about ten seconds.

Secondly, the model performance on distilled datasets is superior. The second graph illustrates the performance of the LightGBM model on the two constructed datasets, with different sample sizes. When using only 50 samples, dataset distillation shows almost no discernible capability. However, with 100 samples, the AUC value of the LightGBM model trained on the distilled dataset reaches approximately 0.876 and further improves with increasing training size. In contrast, the CGAN-generated dataset achieves an AUC of only about 0.5 with 100 samples and stabilizes at a higher value of 0.850 with 2000 samples, but still performs worse than the dataset distilled using the distillation method. It is worthy noting that the synthetic datasets generated from 1000 and 2000 epochs were also demonstrated while there only needs 50 training epochs for the dataset generated from the distilled technique.

We further visualized the distribution of the datasets generated by CGAN and dataset distillation. The results show that the dataset obtained from the distillation method, when decomposed using PCA, has a distribution more similar to the original dataset compared to the CGAN-generated dataset. This explains why the model performance on the distilled dataset is better than that on the CGAN-generated dataset.

**The models’ performance** regarding to a series of metrics are listed in Table 2. We trained each model on the different datasets 5 fold cross validation. Hyper parameters were set as default. As shown in Table 2. We first take a look of the classifiers performance on the original dataset with complete information. when academic information included, all classifiers output acceptable performance. The AUC of Random Forest reaches 0.957, topped among the five classifiers. All models’ performance weakened for predicting student dropout behaviors when the academic performance information was excluded.

**Table 2:** The prediction performance of various models on datasets of with academic performance (left column), and without academic performance (right column), respectively.

Dataset	Complete dataset						Dataset without academic performance					
Metrics	AUC	Std	ACC	Std	F1	Std	AUC	Std	ACC	Std	F1	Std
	**Original dataset**											
Logistic	0.927	0.002	0.889	0.003	0.812	0.005	0.807	0.002	0.773	0.005	0.548	0.005
KNN	0.889	0.006	0.852	0.001	0.728	0.005	0.787	0.004	0.767	0.011	0.580	0.023
RF	0.957	0.000	0.924	0.002	0.876	0.004	0.914	0.001	0.876	0.002	0.785	0.003
LGB	0.892	0.001	0.851	0.003	0.760	0.005	0.742	0.004	0.729	0.003	0.479	0.007
S-LGB	0.912	0.001	0.844	0.001	0.770	0.912	0.794	0.001	0.673	0.040	0.617	0.022
	**Synthetic dataset (conditional generative adversarial networks)**											
Logistic	0.856	0.001	0.813	0.008	0.708	0.009	0.674	0.001	0.611	0.003	0.509	0.008
KNN	0.816	0.005	0.789	0.003	0.656	0.012	0.644	0.008	0.606	0.011	0.505	0.002
RF	0.851	0.004	0.792	0.007	0.694	0.005	0.669	0.002	0.621	0.012	0.486	0.002
LGB	0.838	0.000	0.793	0.001	0.704	0.003	0.634	0.006	0.576	0.004	0.450	0.010
S-LGB	0.843	0.000	0.800	0.002	0.712	0.002	0.637	0.000	0.579	0.001	0.454	0.001
	**Distilled dataset (PP-KIPDD)**											
Logistic	0.918	0.001	0.871	0.001	0.794	0.000	0.795	0.004	0.740	0.008	0.609	0.009
KNN	0.816	0.013	0.784	0.011	0.665	0.008	0.707	0.001	0.675	0.001	0.545	0.009
RF	0.831	0.020	0.412	0.069	0.515	0.027	0.702	0.026	0.357	0.028	0.494	0.006
LGB	0.873	0.001	0.835	0.000	0.746	0.873	0.732	0.010	0.617	0.014	0.571	0.004
S-LGB	0.869	0.000	0.833	0.003	0.745	0.005	0.727	0.009	0.582	0.012	0.565	0.006

*KNN, RF, LGB, S-LGB represents K-Nearest Neighbors, Random Forest, and LightGBM, SMOTE-LightGBM, receptively.

Classifiers trained on synthetic datasets achieved a general lower performance than thant from distilled datasets, seen in Table 2, which corresponding to Fig 5. For the distilled dataset, Logistic Regression, LightGBM and SMOTE-LightGBM yield, in general, good prediction performance in AUC. From this empirical experiment, we did not find the influence of imbalanced distributed classes, this may because the distilled datasets have a balanced distribution. Though there is no single classifier strictly superior, it is worth noting that the model performances on the distilled dataset were close to the raw dataset.

In summary, the reconstruction efficiency of dataset distillation is higher than that of CGAN. When models are trained on the distilled datasets, their performance often surpasses that on CGAN-generated datasets. Since LightGBM has good compatibility with SHAP package, in the following sections, we will apply SHAP values for the explanation analyses of LightGBM on the original datasets with and without academic performance.

### 4.2. Model explanation

#### 4.2.1. Local interpretability analysis.

**Fig 6:**
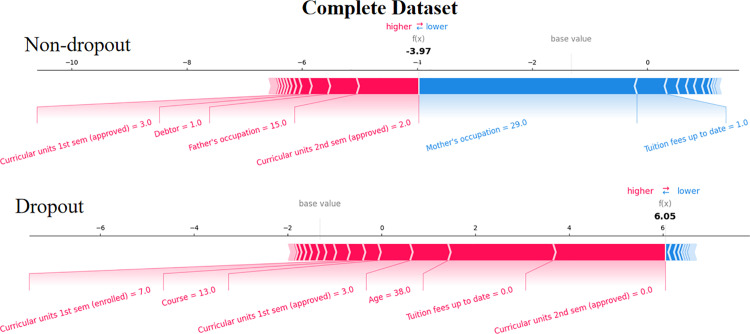
The force plot of 3 students from the datasets including academic information.

**Fig 7:**
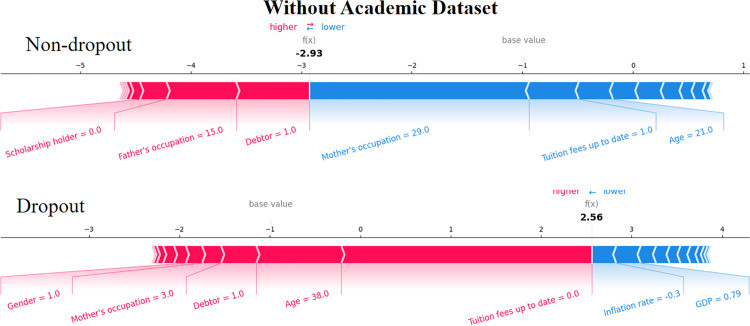
The force plot of 3 students from the datasets without academic information.

The SHAP force plot targets to provide explanations for each individual sample. The following figures show two typical samples. In Figs 6 and 7, the first sample represents student who enrolled or graduated from school courses (in this case, the student status is “enrolled”), while the second sample represents students who dropped out.

As shown in Figs 6 and 7, the red features indicate a positive contribution to the model output, and the blue features are supposed to push the model output. In this study, the student status of complete was set to 0, and the student status of dropout was set to 1. Thus, features with blue push the model prediction towards completing the course while the features in red push the prediction towards a dropout status [25]. The bold values are the model output value (the log-odds) of each prediction [61]. The most contributed features are visible in either red or blue.

In Fig 6, focusing on the first sample, where the student is with non-dropout status, the force plot indicates a model output result of -3.97. The mother’s occupation (with a value of 29, which is the other administrative support staff) and Tuition fee up to date (with a value of 1.0, indicating the tuition fee has been paid) are features contribute most to push the prediction towards a complete status (non-dropout). The curricular units 2nd sem (approved), debtor(with a value of 1.0, indicating the student has a debt), *et al.*, contribute most for push the model prediction towards dropout. As the student’s overall score is less than zero, and the predicted classification is to complete the coursework [62], which aligns with the actual outcome.

A student who dropout has a different force plot, shown in the second row in Fig 6. Curricular units 2nd sem (approved) (with a value of 0, meaning he did not receive any units), tuition fee up to date (with a value of 0, indicating the tuition fee has not been paid), age (a student with 39 year-old), Curricular units 1st sem (approved) (with a low value of 3, compared to the average value of ~ 5.3 for non-dropout student), Course (number is 13, which is the Oral Hygiene), *et al*., push the model prediction towards a dropout status and yield a model output of 6.05, indicating the model regards this student a dropout status, which also aligns with the actual outcome.

We then have a look at the explanation when the academic performance is excluded in Fig 7. In this scenario, the model output result is -2.93. Though the model predicts that this student has completed the coursework which consistent with the actual situation, we see a lowered confidence of the model than when academic information is evaluated (with an output of -3.97, Fig 6). Similar to the prediction with academic information, mother’s occupation, tuition fees up to date contribute most for pushing the model prediction towards the status of complete courses. We also see the feature age is also makes a contribution, this is reasonable since 21 is a common age for student.

The second row in Fig 7 demonstrates the force plot of the student without academic information. Compared to Fig 6, where academic information is evaluated, we saw debtor (with a value of 1, indicating the student has debtor), mother’s occupation (with a value of 3, which is the Specialists in Intellectual and Scientific Activities), and gender (male) are main contributors for pushing the model output towards a dropout status. Inflation rate (-0.3, much lower than the average of the whole sample, which is 1.228 [62]) and GDP (0.79, much higher than the average 0.002 from reference [62]) are main contributors for pushing the model output to a status of complete courses prediction. The overall score of this student is 2.56, which correctly identified the dropout behavior, but demonstrates less confidence to that where academic information is available (Fig 6).

#### 4.2.2. Global interpretability analysis.

**Fig 8:**
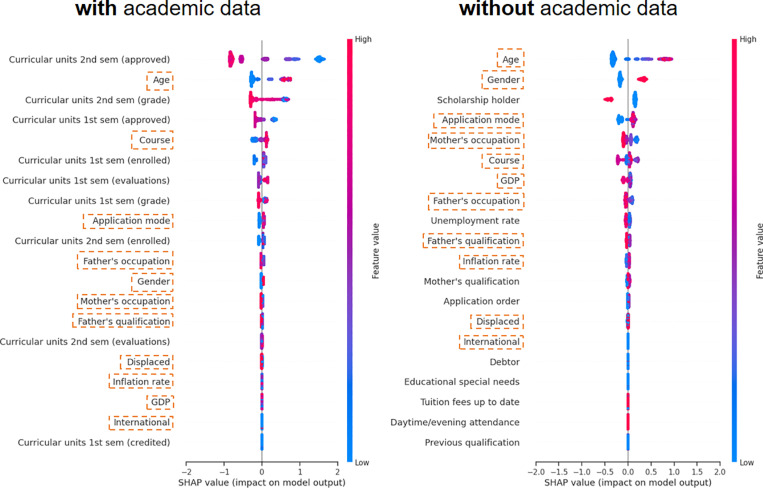
Shows the SHAP value summary plot of most important variables in both datasets. Variables demonstrate red on the left to the y-axis mean a higher value would yield lower risk of dropout, variables with red color on the right side to the y-axis mean a higher value would lead the students with higher risk of dropout. The boxed variables are features showing up their importance in both datasets.

We also visualized the SHAP value summary plot of the two datasets for global interpretability analysis. In Fig 8, the dataset with academic information is shown on the left, while the dataset without academic information is on the right. In our model, students with complete status (enrolled or graduated) are assumed to be completed. Thus, a positive SHAP value represents a student with a high potential risk of dropout, while a negative SHAP value indicates that the student is likely to complete their courses. The red color illustrates higher values of each variable, and the blue color demonstrates lower values.

It can be easily concluded that tuition fees up to date, age, gender, course, application mode, and unemployment rate are essential parameters influencing the classification in both cases. Among them, lower values of age, gender, unemployment rate, and debtor status indicate a student with a low risk of dropout. In other words, young, debt-free female students in an environment with a low unemployment rate and high GDP have a lower dropout risk. We will explain the practicality and significance of these selected variables for educational management in detail in the following discussion.

From the above analysis, it can be seen that the dataset with academic information demonstrates a clearer classification score than the dataset without academic information. However, the dataset without academic information highlights features that determine the classification in a more practical and meaningful way for educational management, such as scholarship information and debt status.

## 5. Discussion

### 5.1. Macroeconomic aspects

According to Becker’s human capital model (HCM), the decision to invest in education is the result of the comparison between expected benefits and costs (both monetary and non-monetary) at the individual level [63,64]. Therefore, the economy has a profound impact on whether students drop out.

In the dataset without academic information, Unemployment rate is the most influential factors in Macroeconomic Category on student dropout behaviors, which demonstrated a negative correlation to dropout risk. When the unemployment rate in the labour market is higher, indicating an increasing number of eligible job seekers who are unable to find a job. This situation introduces a pervasive atmosphere of uncertainty and diminishes the prospects for future employment within the student community, thereby creating an obvious concern among students about their ability to secure meaningful employment upon completing their education. This pervasive uncertainty not only influences individual perspectives but also contributes to a broader social awareness of the intricacies surrounding future career, potentially impacting educational decisions and increasing dropout rates.

GDP is an important factor. Our simulated predictions and SHAP value rankings show that the state of the economy has a profound impact on student dropout rates, and it has been seen here and proven in other research that economic growth and education development are positively correlated [65,66]. This is reasonable because the development of regional or national higher education systems is often viewed as a policy tool that can bring positive economic results in the short and long term [66]. In a better economic environment, graduating from school can lead to better job opportunities and income. Conversely, when the cost of graduation exceeds the expected benefits of obtaining a degree, people seem less interested in completing their studies. Therefore, the relationship between GDP and dropout rates is closely related to the perspectives of economic growth and educational development. This is consistent with many current research viewpoints. For example, Andrei et al. estimated the school dropout rates model depending on the share of expenditures on education in GDP and the number of students per teacher [67–69].

The reason for the low contribution of inflation ratio can be explained by the lag and insensitivity of inflation in schools. For students, inflation usually does not reflect daily life in school because going to school is not a consumption scenario, and inflation usually refers to the rise in goods. However, students have little additional consumption besides tuition fees, so the relationship between student dropout and inflation is small.

### 5.2. Socioeconomic aspect

Qualifications, educational special needs, debtor, tuition fees up to date, and scholarship holder belong to this category. From the SHAP value analysis, tuition fees up to date and scholarship holder are the most important features for discriminating whether a student will complete or drop out of their course, while educational special needs is not as important as the others. In our model, the SHAP value rises as the probability of dropout increases.

The data shows that students with higher tuition fees, younger age, scholarship holders, and lower debt are less likely to drop out of their classes and more likely to complete them. The tuition fees of higher education are always a popular topic in academics, as they relate to local fiscal budgets and the mode of educational reform, such as self-education or student loans. Several studies have shown that the increase in tuition fees might not have a direct influence on the student dropout rate or may even reduce the dropout risk. This is intuitively controversial, but several studies have shown their opinions, such as works by José García Montalvo [70], who claimed there is no evidence of students dropout rate. Steve Bradley [71] believes that the dropout rate will decrease (by 16%). The decrease in dropout rate might be due to the fact that when tuition fees rise, students will re-evaluate their abilities and adjust their expected gains after attending college. Therefore, for students with poor self-assessment abilities, they may drop out early to avoid accumulating debt. It can also be argued that due to the higher costs and higher debts, students with poor abilities are unlikely to enter high-tuition universities. Therefore, due to combination effects, changes in the student population may lead to a decrease in the low dropout rate. This is reasonable, but to more clearly explain why an increase in tuition fees leads to a decrease in dropout rates, it is necessary to further expand the time dimension of the data samples (such as understanding the economic characteristics of incoming students from a more comprehensive perspective). It should be noted that in totally different areas such as rural areas, tuition costs are one of the key factors that determine whether students will continue or drop out of their classes, which are closely linked to their incomes [71,72].

Scholarship holders, or more generally, students who are receiving financial aid, are clearly less likely to drop out of their classes. There are several scenarios. One case is that scholarship holders are students who are comparatively better than their peers, such that they always outperform in academics and have no financial problems. This conclusion agrees with many other studies that demonstrate quantitatively with various datasets (student samples from different locations and periods) that financial aid plays an important role in keeping students from dropping out of their classes [73].

The increase in debt has a negative effect on students who are going to complete their degree, as the increase in debt enhances the cost of graduation and the payments that students need to face when they begin to work off-campus. Various studies have agreed with this finding [74]. Another viewpoint is that students with debt may engage in many behaviors such as part-time work, which may affect their performance in school and increase the risk of being expelled [75]. Therefore, although debt is not one of the most important explainable factors in this dataset, the positive correlation between debt and the dropout probability is also reasonable [76].

### 5.3. Demographic characteristics

Student personal profiles seem to play an important role in the model contribution. Student age is a key factor for students who are clients to dropout or not. SHAP values indicate that younger people are more likely to persist or complete their courses than older ones. This might be due to the fact that although entering college at an older age reduces the return time of higher education, there is reason to believe that if one has a better understanding of their skills and tendencies, the investment cost will also be lower. However, if older students are unlikely to integrate with their peers or interact with professors, the expected non-monetary costs may be higher [63]. Similar conclusions can be found in Luciane Bonaldo’s work [76].

Gender is another controversial issue. We consider the “gender factor” needs to be discussed together with other factors (including but not limited to financial, age, and gender-atypical [34] et al.) to explain its impact on dropout from an educational management perspective. This also explains why the academic community often comes to different conclusions for different sample groups. For example, Francesco Pastore’s study found that gender seems to have little relationship with dropout [63], while Bradley et al. [71] believe that when tuition fees rise, the dropout rate of male students will increase, but female students are not affected. Jasmin Meyer’s empirical analysis concluded that women in gender-atypical subjects show a higher dropout risk than their male fellow students [34], and other studies have quantitatively pointed out that female students have significantly higher dropout rates than male students in science, technology, engineering, and mathematics (STEM) majors [77].

## 6. Conclusions

This article focuses on two key aspects of machine learning applications in education, specifically in predicting student dropout behaviors: privacy concerns and model interpretability. To address these issues, firstly, this study modified the KIPDD protocol and developed a tabular-data-specialized distillation technique-the PP-KIPDD, which showed better performance and higher efficiency compared to the traditional data synthetic technique-the conditional generative adversarial networks. By applying the advantage of PP-KIPDD technique, We establishes a novel framework that employs dataset reconstruction technique to generate synthetic student data, thereby avoiding the leakage of personal information. Secondly, we conducted an interpretability analysis. By analyzing the macroeconomic, socioeconomic, and demographic characteristics of students and correlating them with recent advances in educational research, we provided a detailed study of the selected resampled group. Based on this analysis, we demonstrated that machine learning models can accurately predict student dropout behavior for the selected dataset, and their interpretability (in terms of SHAP values) is explainable from both computational and educational perspectives.

Therefore, introducing a machine learning model combined with dataset distillation techniques is a promising method for addressing student dropout behavior while keep student private information protected. This practical teaching innovation deserves in-depth consideration in the field of sustainable education management. Future work will involve studying different regions and samples to identify more key features in student dropout predictions.
